# Extracts Obtained from *Pterocarpus angolensis* DC and *Ziziphus mucronata* Exhibit Antiplasmodial Activity and Inhibit Heat Shock Protein 70 (Hsp70) Function

**DOI:** 10.3390/molecules22081224

**Published:** 2017-07-28

**Authors:** Tawanda Zininga, Chinedu P. Anokwuru, Muendi T. Sigidi, Milingoni P. Tshisikhawe, Isaiah I. D. Ramaite, Afsatou N. Traoré, Heinrich Hoppe, Addmore Shonhai, Natasha Potgieter

**Affiliations:** 1 Biochemistry Department, School of Mathematical and Natural Sciences, University of Venda, Private Bag X5050, 0950 Thohoyandou, South Africa; tzininga@gmail.com; 2 Chemistry Department, School of Mathematical and Natural Sciences, University of Venda, Private Bag X5050, 0950 Thohoyandou, South Africa; anokwuruchi@gmail.com (C.P.A.); isaiah.ramaite@univen.ac.za (I.I.D.R.); 3 Microbiology Department, School of Mathematical and Natural Sciences, University of Venda, Private Bag X5050, 0950 Thohoyandou, South Africa; muendi.sigidi@yahoo.com (M.T.S.); afsatou.traore@univen.ac.za (A.N.T.); natasha.potgieter@univen.ac.za (N.P.); 4 Botany Department, School of Mathematical and Natural Sciences, University of Venda, Private Bag X5050, 0950 Thohoyandou, South Africa; peter.tshisikhawe@univen.ac.za; 5 Department of Biochemistry and Microbiology, Rhodes University, P.O. Box 94, Grahamstown 6140, South Africa; h.hoppe@ru.ac.za; 6 School of Mathematical and Natural Sciences, University of Venda, Private Bag X5050, 0950 Thohoyandou, South Africa

**Keywords:** antimalarial activity, Hsp70, molecular chaperone, *Pterocarpus angolensis*, *Ziziphus mucronata*

## Abstract

Malaria parasites are increasingly becoming resistant to currently used antimalarial therapies, therefore there is an urgent need to expand the arsenal of alternative antimalarial drugs. In addition, it is also important to identify novel antimalarial drug targets. In the current study, extracts of two plants, *Pterocarpus angolensis* and *Ziziphus mucronata* were obtained and their antimalarial functions were investigated. Furthermore, we explored the capability of the extracts to inhibit *Plasmodium falciparum* heat shock protein 70 (Hsp70) function. Heat shock protein 70 (Hsp70) are molecular chaperones whose function is to facilitate protein folding. *Plasmodium falciparum* the main agent of malaria, expresses two cytosol-localized Hsp70s: PfHsp70-1 and PfHsp70-z. The PfHsp70-z has been reported to be essential for parasite survival, while inhibition of PfHsp70-1 function leads to parasite death. Hence both PfHsp70-1 and PfHsp70-z are potential antimalarial drug targets. Extracts of *P. angolensis* and *Z. mucronata* inhibited the basal ATPase and chaperone functions of the two parasite Hsp70s. Furthermore, fractions of *P. angolensis* and *Z. mucronata* inhibited *P. falciparum* 3D7 parasite growth in vitro. The extracts obtained in the current study exhibited antiplasmodial activity as they killed *P. falciparum* parasites maintained in vitro. In addition, the findings further suggest that some of the compounds in *P. angolensis* and *Z. mucronata* may target parasite Hsp70 function.

## 1. Introduction

Malaria remains a major killer disease with devastating outcomes, particularly in children and pregnant mothers. Although currently malaria is a treatable disease, it is of concern that the population of parasites that are resistant to the most reliable antimalarial treatment (artemisinin-based combination therapies) is growing [1]. It is against this background that it is important to search for alternative effective antimalarial drugs. In addition, it is also important to identify novel antimalarial drug targets.

Heat shock proteins (Hsps) are molecules that facilitate protein folding (molecular chaperones) [2]. The Hsps play a central role in protein quality control and as such they have been proposed as druggable candidates in infectious diseases, among them malaria [3]. The Hsp70 members constitute one of the most ubiquitous molecular chaperone families. Structurally, Hsp70s are constituted by an N-terminal nucleotide binding (ATPase) domain and a substrate binding domain (SBD) located at the C-terminus. These two domains are connected by a linker which facilitates the allosteric signals between the two domains. The Hsp70s exhibit low basal ATPase activity [4]. In the ATP-bound state Hsp70 exhibits low affinity for substrates, leading to substrate release. On the other hand, in the ADP-bound state, Hsp70 possesses higher affinity for substrate binding [5]. The substrate dwell time is influenced by the nucleotides bound to Hsp70s. The Hsp70 functional cycle is regulated by co-chaperones amongst them, Hsp40 proteins. The Hsp40s interact with Hsp70s through their conserved J-domain and activates the Hsp70 ATPase activity [6]. As bi-functional molecules, Hsp40s are also involved in the substrate recruitment to Hsp70.

The Hsp110 proteins are distant family members of Hsp70s, which are constituted by a highly conserved ATPase domain and a less conserved SBD. The Hsp110s have an insertion on the SBD making them larger molecules of average molecular-weight size of 110 kDa, compared to canonical Hsp70s whose sizes are generally around 70 kDa. The Hsp110 proteins function as nucleotide exchange factors (NEF) for canonical Hsp70s [7].

The main agent of malaria, *Plasmodium falciparum*, expresses six members of the Hsp70 family of which two are cytosol-localized: PfHsp70-1 and PfHsp70-z [8]. Parasite Hsp70s have been proposed as potential antimalarial drug targets [3,9]. We previously reported that both PfHsp70-1 and PfHsp70-z exhibit independent chaperone functions [10,11,12,13,14]. The PfHsp70-1 is a canonical Hsp70, as it is closely related to the *Escherichia coli* Hsp70 homolog (DnaK). On the other hand, PfHsp70-z belongs to the Hsp110 family. The Hsp110s exhibit independent chaperone activity apart from serving as NEFs of canonical Hsp70s [7]. We previously reported that PfHsp70-1 interacts with PfHsp70-z, and for this reason we think that PfHsp70-z acts as an independent molecular chaperone and may also serve as a NEF for PfHsp70-1 [13]. The two proteins are thought to function in cooperation towards facilitating protein folding to facilitate survival of malaria parasites. Protein quality control is important for the survival of the parasite, since 24% of the *P. falciparum* proteome is composed of asparagine repeat rich proteins which tend to aggregate under stress [15]. Both PfHsp70-z and PfHsp70-1 are thought to play a crucial role under the physiologically stressful conditions the parasites encounter during their life cycle. PfHsp70-1 and PfHsp70-z are particularly important at the blood stage during the development of clinical malaria [16,17].

*Pterocarpus angolensis* DC belongs to the family of Fabaceae [18]. *P. angolensis* has been reported to have antimicrobial properties and is used to treat malaria, among other diseases [18,19,20,21]. *Ziziphus mucronata* belongs to the Rhamnaceae family and is found in most parts of South Africa [22]. Stem and bark infusions have previously been reported to have antimicrobial activity [23,24].

Heat shock proteins have been proposed as antimalarial drug targets [9,11] and thus elucidating their inhibitors presents an alternative option towards antimalarial drug discovery. We recently demonstrated inhibition of parasite Hsp70 function by the cyclic peptide antibiotic, polymyxin B [14]. As plants contain a wide range of potential small molecule inhibitors of proteins, we speculated that some of the inhibitors may target heat shock protein function.

We investigated the effects of the *P. angolensis* and *Z. mucronata* extracts on the functional features of both PfHsp70-1 and PfHsp70-z. In addition, we investigated the effects of the *P. angolensis* and *Z. mucronata* extracts on the viability of *P. falciparum* 3D7 parasites maintained at the blood stage. Data from this study demonstrate that select fractions from the *P. angolensis* and *Z. mucronata* extracts inhibit Hsp70 chaperone function. Furthermore, our findings established that the *P. angolensis* and *Z. mucronata* fractions possess antiplasmodial activity. We discuss the implications of our findings and the prospects of further characterizing the compounds in *P. angolensis* and *Z. mucronata* extract fractions as inhibitors of Hsp70 function in malaria.

## 2. Results

### 2.1. Z. mucronata and P. angolensis Extracts Contain Phenolic Compounds

We quantified the phenolic compounds in the *P. angolensis* and *Z. mucronata* fractions using mass spectrometric (MS) analysis (Supplementary Table S1). The MS analysis showed that ZF2 contains the highest phenolic content (Table 1). In addition, both *P. angolensis* and *Z. mucronata* fractions contain epicatechin, and low levels of gallic acid, taxifolin and rutin (Table 1).

### 2.2. Z. mucronata and P. angolensis Extracts Suppress the Chaperone Function of Both PfHsp70-1 and PfHsp70-z

We expressed and purified both recombinant PfHsp70-1 and PfHsp70-z from *E. coli* XL1 Blue and *E. coli* JM109 cell lines, respectively (Supplementary Figure S1). Using the recombinant forms of both PfHsp70-1 and PfHsp70-z, we previously showed that they are heat stable and are capable of suppressing the heat induced aggregation of model substrates such as MDH [10,13,14,25]. In a previous study, we observed that the chaperone activity (suppression of heat induced protein aggregation) of PfHsp70-z was not influenced by nucleotides while ATP inhibited the chaperone activity of PfHsp70-1, respectively [10,13]. In the current study, MDH was subjected to heat stress at 48 °C, and as expected it did aggregate in the absence of chaperones (Table 2).

In the presence of a non-chaperone protein (BSA), MDH also aggregated in response to heat stress. It was important to validate that the solvent used 0.01% DMSO did not affect the stability for the chaperones, and for this reason it was used as baseline control. When the chaperones were excluded from the reaction mixture, only MDH fully aggregated in the presence and absence of 25 µg/mL *Z. mucronata*/*P. angolensis* extracts. However, at the same level (25 µg/mL) of *Z. mucronata*/*P. angolensis* extracts did not influence the solubility of either PfHsp70-1 or PfHsp70-z subjected to heat stress. Assessment of the thermal stability of PfHsp70-1/PfHsp70-z/BSA/MDH was conducted by monitoring the heat induced aggregation of the respective protein in vitro at 48 °C. The degree of aggregation was estimated by monitoring the increase in optical density using spectroscopy at 320 nm. Relative aggregation was normalized to spontaneous MDH aggregation. Standard deviations obtained from three replicate assays are shown.

**Figure 1:**
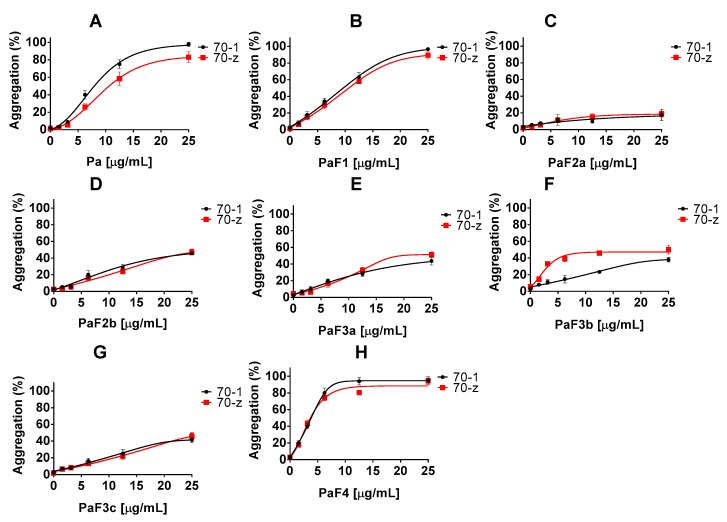
*P. angolensis* extracts suppress chaperone function of PfHsp70-1 and PfHsp70-z. The chaperone function of PfHsp70-1 and PfHsp70-z was investigated by monitoring the heat induced aggregation of MDH in the presence of PfHsp70-1/PfHsp70-z (chaperone) in vitro at 48 °C for 60 min. The resultant heat induced aggregation of MDH was estimated by taking readings at 360 nm. The values were normalized to the aggregation of spontaneous MDH aggregation in the absence of PfHsp70-1 (70-1)/PfHsp70-z (70-z). The assay was repeated in the presence of the various plant extracts and represented by various panels: (**A**) Pa; (**B**) PaF1; (**C**) PaF2a; (**D**) PaF2b; (**E**) PaF3a; (**F**) PaF3b; (**G**) PaF3c; (**H**) PaF4. Standard deviations obtained from three replicate assays are shown.

In the presence of *P. angolensis* crude extract, Pa and the fractions PaF1 and PaF4, the chaperone activities of either PfHsp70-1 or PfHsp70-z were inhibited in a concentration dependent manner (Figure 1, Table 3). However, the *P. angolensis* fractions (PaF2a/b; PaF3a/b/c) did not suppress the activities of the two proteins (Figure 1).

**Figure 2:**
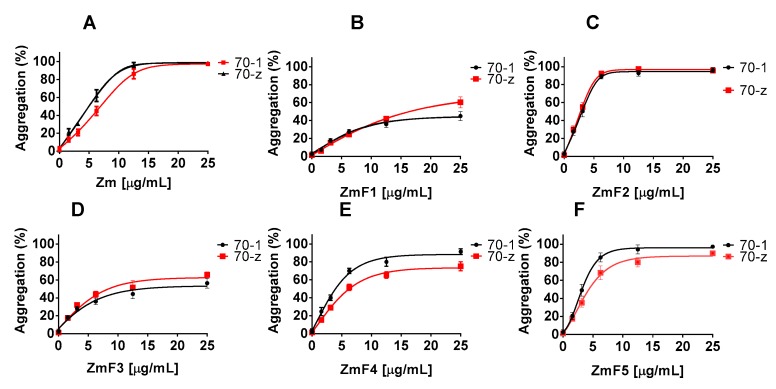
Inhibition of the chaperone activities of PfHsp70-1 and PfHsp70-z by *Z. mucronata* extracts. Heat induced aggregation of MDH was monitored in the presence and absence of PfHsp70-1/PfHsp70-z (70-1/70-z) at 48 °C for 60 min. The resultant aggregates were estimated at 360 nm. The values were normalized to aggregation of spontaneous MDH aggregation in the absence of PfHsp70-1/PfHsp70-z. Data obtained in the presence of various plant extracts is provided: (**A**) Zm; (**B**) ZmF1; (**C**) ZmF2; (**D**) ZmF3; (**E**) ZmF4; (**F**) ZmF5. Standard deviations obtained from three replicate assays are shown.

Similarly, the crude extract of *Z. mucronata* (Zm) and fractions ZmF2, ZmF4 as well as ZmF5 suppressed the chaperone function of both PfHsp70-1 and PfHsp70-z in a concentration dependent manner (Figure 2, Table 3). However, fractions ZmF1 and ZmF3 were not effective at suppressing the chaperone function of both proteins.

### 2.3. Select P. angolensis and Z. mucronata Extracts Inhibit PfHsp70-1 and PfHsp70-z ATPase Activity

**Figure 3:**
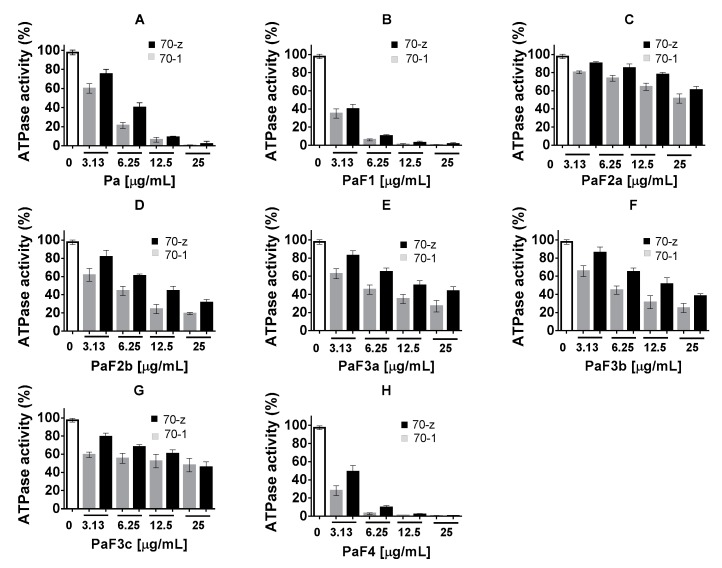
*P. angolensis* extracts inhibit the basal ATPase activity of PfHsp70-1 and PfHsp70-z. The basal ATPase activities of PfHsp70-1 (70-1) and PfHsp70-z (70-z) were analysed in the presence of various plant extracts. The values were normalized to the basal ATPase activities of each protein obtained in the absence of plant extract. The effects of plant extract under variable concentrations were then investigated and represented: (**A**) Pa; (**B**) PaF1; (**C**) PaF2a; (**D**) PaF2b; (**E**) PaF3a; (**F**) PaF3b; (**G**) PaF3c; (**H**) PaF4. Standard deviations obtained from three replicate assays are shown.

Previous studies have shown that PfHsp70-z and PfHsp70-1 both bind to ATP with comparable affinities. Furthermore, both proteins exhibit ATPase activities within the same range of magnitude [13]. In the current study, the ATPase activities of both Hsp70s were determined under ATP saturating levels (5 mM), while the concentration of *P. angolensis* was varied (0–25 µg/mL) (Figure 3). We noted that the ATPase activities of the respective protein decreased with increase in the concentration of fractions Pa, PaF1, PaF4 (Figure 3). This suggests that the fractions contain compounds that specifically interfered with the ATPase function of both PfHsp70-1 and PfHsp70-z. On the other hand, fractions PaF2a/b, PaF3a/b/c, were generally ineffective (Figure 3).

**Figure 4:**
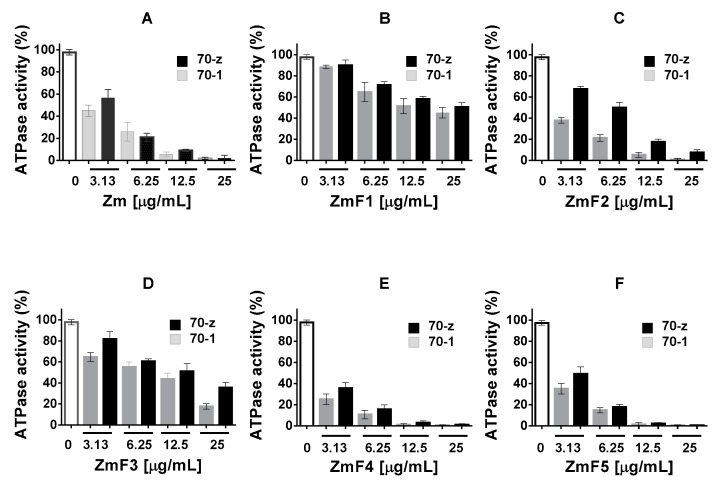
Inhibition of the basal ATPase activity of PfHsp70-1 and PfHsp70-z by *Z. mucronata* extracts. The basal ATPase of PfHsp70-1 (70-1)/PfHsp70-z (70-z) were analysed in the presence of increasing amounts of the various plant extracts. The values were normalized to basal ATPase of each protein obtained in the absence of plant extract. The effects of the variable concentrations of plant extracts were then investigated and presented: (**A**) Zm; (**B**) ZmF1; (**C**) ZmF2; (**D**) ZmF3; (**E**) ZmF4; (**F**) ZmF5. Standard deviations obtained from three replicate assays are shown.

The ATPase assay was repeated in the presence of varying amounts (up to a maximum of 25 µg/mL) of the *Z. mucronata* extract (Figure 4). As the concentration of crude extract Zm and fractions ZmF2, ZmF4, ZmF5 increased, the ATPase activities of the two proteins were suppressed. This again suggests that these fractions contain compounds that inhibit the ATPase activity of Hsp70. On the other hand, fractions ZmF1 and ZmF3 did suppress the ATPase activities of the two proteins, albeit less effectively (Figure 4).

### 2.4. Z. mucronata and P. angolensis Extracts Inhibit Growth of Plasmodium falciparum Parasites

Growth inhibition assays were conducted on *P. falciparum* 3D7 maintained at the blood stage based on the *P. falciparum* lactate dehydrogenase (pLDH) method as previously described [26]. First it was important to validate the antimicrobial sensitivity of the parasites. For this reason, 1 µM chloroquine, a known antimalarial drug that inhibits *P. falciparum 3D7* growth [27], was used as a positive control. Furthermore, DMSO was used within similar range (0.05%) as previously recommended to allow maximum parasite viability [28]. *P. falciparum 3D7* cells cultured in the absence of extracts were used as negative controls, and these as expected exhibited maximum viability. The treatment of parasite cultures with 25 µg/mL of *P. angolensis*/*Z. mucronata* extracts resulted in the inhibition of parasite growth. The crude extracts Pa, Zm as well as fractions PaF1, PaF4, ZmF2 and ZmF5 inhibited parasite growth by more than 75%. In addition, the fractions that inhibited 75% of parasite growth were further analysed to determine the concentration that suppressed parasite growth by up to 50% (IC_50_). As a limitation, for some extracts the concentration range used was not high enough to obtain 0% viability. *P. falciparum* 3D7 cell line is a known chloroquine sensitive strain and in the current study chloroquine registered an IC_50_ of 8.5 ng/mL which is within the reported range from previous studies (Table 4, [27]). We observed that *P. angolensis* fractions 1 and 4 both exhibited significantly lower IC_50_ values in comparison with *Z. mucronata* fractions 2 and 5 (*p* < 0.05, Table 4). This suggests that *P. angolensis* contain compounds with more potent antiplasmodial properties than *Z. mucronata*.

## 3. Discussion

The emergence of multidrug resistance by *P. falciparum* parasites has resulted in a high unmet need for novel antimalarial drugs to combat malaria. This study provides the first direct evidence of the anti-chaperone function and antiplasmodial activity of extracts from both *Z. mucronata* and *P. angolensis* plants. Our findings suggest that the two plants contain compounds that inhibit parasite growth in vitro. In addition, some of the plant compounds have the capability to suppress the ATPase and chaperone functions of two prominent *P. falciparum* chaperones, PfHsp70-1 and PfHsp70-z which are localized to the parasite cytosol [13]. We previously observed that compounds that inhibit PfHsp70-z and PfHsp70-1 such as polymyxin B [14] and (−)-epigallocatechin-3-gallate (EGCG, unpublished data) also inhibit parasite growth. In addition, such small molecule inhibitors also abrogated association of the two chaperones with their functional partners [14]. It is therefore possible that both *Z. mucronata* and *P. angolensis* plants contain compounds that interfere with either ATP binding by Hsp70 as well those that interfere with capacity of the chaperone to bind substrates. Although the fractions derived here exhibited higher IC_50_ than chloroquine, they showed promising antimalarial activity. It is also possible that the plant extracts may have inhibited other functions besides the protein folding pathway of the parasite, in exerting their antiplasmodial activity.

The plant extracts used in the current study were fractioned using increasing polarity of the respective solvents. It is well known that different compounds are fractioned under various solvents polarity. For example, it has been previously reported that hexane generated fractions lack antimalarial activity [29,30,31], which is in line with the current results as ZmF1 which lack phenolic compounds, did not exhibit significant anti-chaperone nor anti- malarial activities, respectively (Figure 2 and Figure 4, Table 1 and Table 2).

Hexane fractions have been shown to comprise phenols, triterpenoids, terpenoids and tannins [29,31]. Interestingly, results from this study showed that fraction PaF1 (50% ethyl acetate and 50% hexane) exhibited potent antiplasmodial activity (Table 3). This suggests that despite the hexane derived ZmF1 fraction lacking antimalarial activity, increasing the ethyl acetate component to 50% (fraction PaF1) led to the recovery of compounds that do possess antimalarial activity. However, the antiplasmodial activity was not only a function of the solvent used to extract the products, but was also dependent on the plant source (Table 3).

Ethyl acetate fractions generally contain phenols, triterpenoids, terpenoids, flavonoids and steroids [30,31]. Of these, flavonoids and phenols have been previously reported to possess potent antimalarial activity [29,31]. This composition possibly explains the antiplasmodial activity observed in this study. Methanol extracts have been previously reported to contain a variety of compounds [32]. It would therefore be important to establish the compounds contained in fractions ZmF5 and PaF4 that exhibit antiplasmodial activity.

One of the main pathways targeted by most antimalarial compounds is the oxido-reductory system [33,34]. In the current study, we explored the protein folding pathway as a potential antiplasmodial drug target. This is because heat shock proteins have been proposed as prospective antimalarial drug targets [3,9]. However, heat shock proteins tend to be conserved, thereby confounding their suitability as antimalarial drug targets. One of the validated targets of the extracts derived in the current study is the essential, cytosol localized parasite heat shock protein, PfHsp70-z [8,13]. The PfHsp70-z belongs to Hsp110 subfamily which generally exhibit very low sequence conservation compared to its human equivalence [13]. Hence, the observed inhibition of PfHsp70-z by the compounds isolated from this study is important towards antiplasmodial drug design. In addition, *P. falciparum* proteome is estimated to be comprised of asparagine repeat proteins which constitute at least 25% of the population asparagine repeat proteins are generally aggregation prone. The PfHsp70-z has been reported to possess independent chaperone function and the protein is deemed to play an important function in regulating protein quality control, especially buffering aggregation prone proteins of the parasite against cellular stress [13].

Polyphenols were generally present in all the fractions irrespective of the extraction solvents used, except fraction PaF2a which exhibited significantly lower polyphenol content (Table 1) [35]. Previous studies have shown that flavonoids are extracted effectively using ethyl acetate [29]. The PaF1 was the only fraction with a C=O in the IR region of phenolic esters. This could represent saponins (i.e., terpenoids with glycosides) or flavonoids with glycosides. The PaF1 did not display any antioxidant activities and further analysis using MS confirmed that it lacks the phenolic compounds [35]. However, the presence of the alkyl group in the IR data could signify the presence of terpenoids which is consistent with the potent antiplasmodial activity that it exhibited (Table 4) [35]. In addition, extracts F2 and F4 from *Z. mucronata* obtained using a solvent rich in ethyl acetate showed strong antiplasmodial activity (Table 1). This observation is in line with previous studies which demonstrated that green tea plant extract and husk extract (fractions of *Zea mays*) obtained using ethyl acetate exhibited notable antiplasmodial function [29,31].

Altogether, the current study provides the first evidence for the antiplasmodial activity for *P. angolensis* and *Z. mucronata* stem bark extractions. In addition, some of the obtained fractions inhibited the chaperone functions of two cytosol localized parasite Hsp70 homologues. The findings suggest that amongst the compounds present in the extracts some target Hsp70 function. For example, notable in some of the fractions are catechins whose antimalarial activity has also been reported [29]. We previously showed that compounds that inhibit parasite Hsp70, tend to not only inhibit the chaperone function of the protein but abrogate association of Hsp70 with its functional associates [14]. This makes inhibition of parasite Hsp70 an attractive drug target. There is need to further characterize the fractions obtained here in order to identify compounds that are responsible for the observed antiplasmodial activity towards expanding the drug arsenal against malaria. This is especially important in the wake of drug resistance being increasingly mounted by malaria parasites.

## 4. Materials and Methods

### 4.1. Materials

Unless, specified chemical reagents used in this study were purchased from Merck (Darmstadt, Germany), Sigma Aldrich (St. Louis, MO, USA), and Melford (Suffolk, UK).

### 4.2. Fractionation of Pterocarpus angolensis and Ziziphus mucronata Stem Bark Extracts

The plants were collected by Muendi Sigidi in the Vuwani area (Hamangilasi, Vhembe District Municipality of Limpopo Province, South Africa) on the 28th of January 2015. The voucher specimen number for *Pterocarpus angolensis* is MPT00118. The voucher specimen number for *Ziziphus mucronata* is MPT00123 and the plant was collected at Mukomawabani (Tshikondeni, Vhembe District Municipality) in 2014. The two plants were identified using their Venda vernacular names, Makhalu and Mutondo in their natural habitat in the Vhembe District of Limpopo Province. The plants were later confirmed by the taxonomic rank at the Department of Botany, University of Venda with reference to the international plant name index (*Ziziphus mucronata* Willd [MPT00123] and *Pterocarpus angolensis* DC [MPT00118] as previously described [21]. Stem bark samples were processed for each plant, the samples were first air dried and ground to powder using a Büchi mixer (Büchi Labortechnik AG, Flawil, Switzerland). The samples of *P. angolensis* bark were soaked as 5% (*w*/*v*) to solvent 1 (50% dichloromethane and 50% methanol) at room temperature for 24 h. Then, the extract was filtered and evaporated with a rotor vapor at 40 °C to obtain the crude extract (Pa). The crude extract was fractioned using a silica gel 60 column chromatography and was eluted with hexane and increasing polarity with ethyl acetate and finally methanol to obtain 7 fractions as previously described [35]. Briefly, *P. angolensis* fraction 1 (PaF1) was obtained from 50% hexane, 50% ethyl acetate (1:1), PaF2a and 2b were obtained from 90% ethyl acetate, 10% methanol; PaF3a-c were obtained from 70% ethyl acetate and 30% methanol while PaF4 was obtained from 10% ethyl acetate 90% methanol [35]. The stem bark for *Ziziphus mucronata* (MPT00123) was also processed as previously described [21] and used to extract fractions by soaking in ethanol to obtain the crude extract (Zm). The Zm was also subjected to silica gel column chromatography and eluted in five fractions. *Z. mucronata* fraction 1, (ZmF1) was eluted with 70% hexane and 30% ethyl acetate; fraction 2 (ZmF2) and fraction 3 (ZmF3) were eluted with 90% ethyl acetate and 10% methanol; fraction 4 (ZmF4) was eluted with 70% ethyl acetate and 30% methanol and fraction 5 (ZmF5) was eluted with 30% ethyl acetate and 70% methanol.

### 4.3. Quantification of Phenolics in Pterocarpus angolensis and Ziziphus mucronata Extracts

The presence of caffeic acid, catechin, epicatechin, gallic acid, protocatechuic acid, taxifolin and rutin was determined in the fractions of *P. angolensis* and *Z. mucronata* using a UPLC-QToF-MS method developed by the Central Analytical Facilities (CAF), University of Stellenbosh, South Africa. The method was validated for linearity and precision using standard procedures [36,37]. A concentration range of 1–100 μg/mL was used to construct the calibration curves for, caffeic acid, gallic acid, protocatechuic acid, and taxifolin, while a concentration range of 3.5–111 μg/mL was used to construct calibration curves for catechin, epicatechin, and rutin, respectively. The phenolics were identified and quantified by comparing the retention time of the standards with the samples analysed (Supplementary Table S1). A Synapt G2 quadrupole time-of-flight mass spectrometer (Waters, NewYork, NY, USA) was used for LC-MS analysis. It was fitted with a Waters Ultra pressure liquid chromatograph and photo diode array detection. Separation was achieved on a Waters BEH C18, 2.1 × 100 mm column with 1.7 µm particles. A gradient was applied using 0.1% formic acid (solvent A) and acetonitrile containing 0.1% formic acid (solvent B). The gradient started at 100% solvent A for 1 minute and changed to 28% B over 22 min in a linear way. It then went to 40% B over 50 s and a wash step of 1.5 min at 100% B, followed by re-equilibration to initial conditions for 4 min. The flow rate was 0.3 mL/min and the column was kept at 55 °C. The injection volume was 2 µL. Data was acquired in MSE mode which consisted of a low collision energy scan (6V) from *m*/*z* 150 to 1500 and a high collision energy scan from *m*/*z* 40 to 1500. The high collision energy scan was done using a collision energy ramp of 30–60 V. The photo diode array detector was set to scan from 220 to 600 nm. The mass spectrometer was optimized for best sensitivity, a cone voltage of 15 V, desolvation gas was nitrogen at 650 L/h and desolvation temperature 275 °C. The instrument was operated with an electrospray ionization probe in the negative mode. Sodium formate was used for calibration and leucine encephalin was infused in the background as lock mass for accurate mass determinations.

### 4.4. Expression and Purification of Recombinant Proteins

A construct expressing PfHsp70-1 (pQE30/PfHsp70-1 [10], and PfHsp70-z pQE30/PfHsp70-z [11]) were used for the expression of recombinant PfHsp70-1 and PfHsp70-z proteins using *E. coli* XL1 Blue and JM109 cells respectively, following a previously described method [10,11]. The recombinant proteins were purified using affinity chromatography as previously described [13].

### 4.5. Analysis of the Effect of P. angolensis and Z. mucronata Extracts on the Chaperone Function of PfHsp70-1 and PfHsp70-z

The effects of *P. angolensis* and *Z. mucronata* extracts on the chaperone function of both PfHsp70-1 and PfHsp70-z were investigated by monitoring the heat-induced aggregation of a model protein, malate dehydrogenase (MDH) from *Photinus pyralis* (Sigma-Aldrich, Saint Louis, MO, USA) as previously described [13] with minor modifications. In order to determine the effects of different plant extracts on the chaperone function of both PfHsp70-1 and PfHsp70-z, the assay was conducted in the presence of varying amounts of the plant extract (0–50 µg/mL). The aggregation reaction was monitored at 320 nm for 1 h and data was processed using GraphPad prism 6 (San Diego, CA, USA).

### 4.6. Investigation of the Effects of P. angolensis and Z. mucronata Extracts on Basal Hsp70 ATPase Activity

The basal ATPase activities of PfHsp70-1 and PfHsp70-z Hsp70 were determined by a colorimetric method as previously described [13,14]. In order to determine the effects of *P. angolensis* and *Z. mucronata* extracts on the basal ATPase activity on parasite Hsp70s, the assay was repeated in the presence of varying amounts (0.1–50 µg/mL) of either *P. angolensis* and *Z. mucronata* extract. As controls, the respective boiled Hsp70 protein was used to cater for spontaneous hydrolysis of ATP.

### 4.7. Investigation of the Effects of P. angolensis and Z. mucronata Extracts on P. falciparum Parasite Growth

*P. falciparum* 3D7 parasites were grown and maintained in continuous culture as previously described [12,38]. The *P. falciparum* lactate dehydrogenase (pLDH) method was used to monitor the growth of *P. falciparum* 3D7-infected erythrocytes subjected to different amounts of (0.01–25 µg/mL) *P. angolensis* and *Z. mucronata* extracts treatments as previously described [26]. Initial growth assay screening was conducted at 25 μg/mL plant extract concentration. The plant extracts with 75% growth inhibition of parasite were further analysed for IC_50_ determination by titrating (range 0.001–20 μg/mL) the plant extract concentration. Analysis of the pLDH data for growth inhibition assay was carried out using GraphPad Prism 6. All experiments in this study were repeated at least three times with three runs each time.

## 5. Conclusions

We demonstrated that extracts of both *P. angolensis* and *Z. mucronata* exhibit antiplasmodial activity. Crude extracts of the two plants and some of their respect fractions (PaF1, PaF4, ZmF2, ZmF4 and ZmF5) effectively suppressed the chaperone activities and ATPase functions of the two parasite cytosol localized chaperones (PfHsp70-1 and PfHsp70-z). This suggests that the plants contain compounds that may abrogate ATP binding by Hsp70, and at the same time, the compounds interfere with the capacity of the chaperones to bind their substrates (misfolded proteins). Furthermore, that fractions, PaF1, PaF4, ZmF2, and ZmF5 also further inhibited parasite growth provides evidence that the plant products abrogate Hsp70 function to inhibit parasite growth. However, it cannot be excluded that some of the compounds from the plants may target other pathways in the parasite. It is important to further establish the exact compounds implicated in the processes leading to parasite death.

## Figures and Tables

**Table 1:** Quantification of phenolic compounds in *Z. mucronata* and *P. angolensis* extracts.

Samples	Compounds [mg/g Dry Weight]/(±Standard Deviation; *n* = 3)						
	Protocatechin	Catechin	Gallic Acid	Caffeic Acid	Rutin	Epicatechin	Taxifolin
ZF1	ND	-	-	-	-	-	-
ZF2	0.04 (±0.003)	2.25 (±0.12)	-	0.29 (±0.02)	0.02 (±0.001)	5.34 (±0.08)	0.16 (±0.01)
ZF3	0.26 (±0.02)	1.71 (±0.21)	-	2.72 (±0.20)	-	4.06 (±0.30)	0.09 (±0.01)
ZF4	0.46 (±0.03)	0.72 (±0.16)	0.41 (±0.03)	-	-	1.47 (±0.02)	0.04 (±0.002)
ZF5	0.58 (±0.04)	0.23 (±0.04)	0.18 (±0.02)	-	-	0.41 (±0.01)	0.01 (±0.001)
PaF1	ND	-	-	-	-	-	-
PaF2a	ND	-	--	-	-	15.1 (±0.23)	0.02 (±0.001)
PaF2b	ND	-		-	-	0.19 (±0.001)	-
PaF3a	ND	-	-	-	-	0.02 (±0.003)	-
PaF3b	ND	-	-	-	-	0.41 (±0.006)	-
PaF4	ND	-	-	-	-	0.05 (±0.0001)	-

Legend: ND-not detected; Standard deviations are for intraday variation.

**Table 2:** Comparative thermal stability of PfHsp70-1, PfHsp70-z and MDH in the presence of *P. angolensis* and *Z. mucronata* extracts.

	Relative Aggregation% (±Standard Deviation; *n* = 3)									
	Buffer	DMSO	Pa	PaF1	PaF2a	PaF2b	PaF3a	PaF3b	PaF3c	PaF4
PfHsp70-1	1.2 (±0.9)	1.3 (±0.5)	2.3 (±0.5)	1.2 (±0.9)	1.4 (±0.3)	3.3 (±0.4)	1.3 (±0.5)	1.6 (±0.9)	1.9 (±0.7)	2.2 (±0.8)
PfHsp70-z	1.1 (±0.8)	2.2 (±0.2)	1.2 (±0.4)	1.9 (±0.8)	3.2 (±0.5)	1.3 (±0.2)	1.2 (±0.8)	2.0 (±0.6)	1.2 (±0.2)	1.7 (±0.8)
BSA	1.6 (±1.2)	1.2 (±0.2)	1.5 (±0.9)	1.6 (±0.4)	1.3 (±0.5)	1.2 (±0.9)	1.9 (±0.7)	2.7 (±1.4)	3.9 (±2.7)	1.6 (±1.2)
MDH	98.0 (±1.2)	91.6 (±1.2)	86.6 (±1.2)	97.2 (±0.9)	91.6 (±1.2)	90.2 (±0.9)	89.6 (±1.2)	95.2 (±0.9)	98.6 (±1.2)	92.2 (±0.9)
	**Buffer**	**DMSO**	**Zm**	**ZmF1**	**ZmF2**	**ZmF3**	**ZmF4**	**ZmF5**		
PfHsp70-1	1.2 (±0.9)	1.3 (±0.5)	1.6 (±0.2)	1.4 (±0.3)	1.5 (±0.6)	1.2 (±0.9)	1.9 (±0.3)	1.7 (±0.7)		
PfHsp70-z	1.1 (±0.8)	1.2 (±0.8)	1.7 (±0.4)	1.6 (±0.8)	1.9 (±0.3)	1.7 (±0.1)	1.5 (±0.2)	1.8 (±0.8)		
BSA	1.6 (±1.2)	1.3 (±0.2)	2.0 (±0.9)	1.7 (±0.4)	1.5 (±0.5)	1.6 (±0.9)	2.0 (±0.7)	2.2 (±1.7)		
MDH	98.3 (±1.2)	90.6 (±1.2)	97.2 (±0.2)	89.5 (±0.9)	93.6 (±0.4)	91.3 (±0.5)	95.2 (±0.9)	92.9 (±0.7)		

**Table 3:** Comparative IC_50_ inhibition of *P. angolensis* and *Z. mucronata* extracts on PfHsp70-1 and PfHsp70-z chaperone activities.

	IC_50_ (µg/mL)/[±Standard Deviation]									
	Pa	PaF1	PaF2a	PaF2b	PaF3a	PaF4	Zm	ZmF2	ZmF4	ZmF5
PfHsp70-1	12.3 (±1.2)	0.5 (±0.2)	ND	15.6 (±2.1)	10.1 (±1.5)	0.8 (±0.3)	9.3 (±1.8)	3.1 (±1.1)	ND (±5.2)	6.0 (±1.2)
PfHsp70-z	17.5 (±2.1)	9.0 (±1.1)	6.5 (±0.2)	ND	13.7 (±2.0)	6.1 (±1.0)	13.8 (±2.1)	4.3 (±0.4)	3.2 (±1.0)	6.4 (±0.3)

**N**D—undetermined (>25 µg/mL), PaF3b, PaF3c, ZF1 and ZF3 were not tested. Standard deviations obtained from three replicate assays are shown.

**Table 4:** IC_50_ values for the antiplasmodial activities of *P. angolensis* and *Z. mucronata* extracts

Compound	IC_50_ (µg/mL)/[±Standard Deviation; *n* = 2]
Pa	13.87 (±0.20)
PaF1	0.7945 (±0.002)
PaF4	1.961 (±0.01)
Zm	7.4 (±0.3)
ZmF2	6.404 (±0.02)
ZmF5	19.9 (±0.3)
Chloroquine	0.008522 (±0.0004)

## Supplementary Materials

The following are available online; Figure S1: Expression purification of recombinant PfHsp70-1 and PfHsp70-z; Table S1: The linear range, correlation coefficient, LOD and LOQ of each standard used in quantification of phenolics.
