# Supplementary material for: Extracts Obtained from Pterocarpus angolensis DC and Ziziphus mucronata Exhibit Antiplasmodial Activity and Inhibit Heat Shock Protein 70 (Hsp70) Function
Source: Molecules. 2017 Jul 28;22(8):1224. doi: 10.3390/molecules22081224 (PMC6152082; doi:10.3390/molecules22081224)
Supplement: Supplementary file 1 [file molecules-22-01224-s001.pdf]

Supplementary Table 1. The linear range, correlation coefficient, LOD and LOQ of each standard used to quantify the phenolics

Standards	Coefficient of Correlation (R^2)	Linear range (µg/mL)	LOD (µg/mL)	LOQ (µg/mL)	RSD Intra day (%)	RSD Inter day (%)
Caffeic acid	0.990	1-100	0.33	1.0	8.0	18
Catechin	0.998	3.5-111	0.33	1.0	0.9	5.7
Gallic acid	0.997	1-100	0.33	1.0	9.3	22
Epicatechin	0.998	3.5-111	0.33	1.0	1.5	8.3
Protocatechuic acid	0.993	1-100	0.33	1.0	7.0	15.7
Rutin	0.990	3.5-111	0.17	0.50	4.0	16.5
Taxifolin	0.997	1-100	0.83	2.50	6.0	17.3

LOD: limit of detection; LOQ: limit of quantification, RSD: relative standard deviation (n = 3)

Supplementary Figure

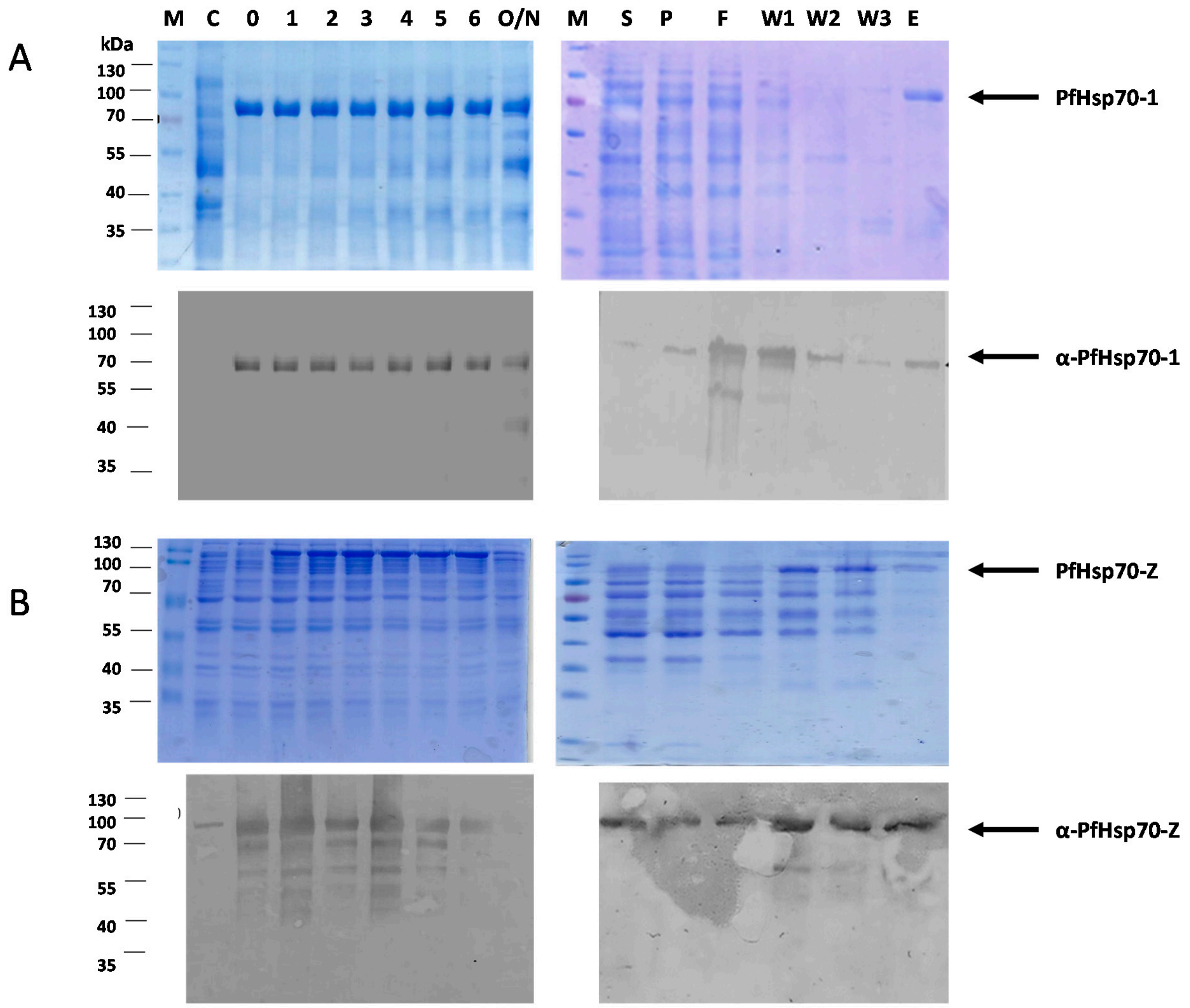

Figure S1: Expression purification of recombinant PfHsp70-1 and PfHsp70-z. (A) PfHsp70-1 was expressed in *E. coli* XL1 Blue cells transformed with pQE30/PfHsp70-1. SDS-PAGE (12 %) and Western analyses (lower panel) of the expression of recombinant PfHsp70-1, the purification of PfHsp70-1 as previously described [14]. (B) PfHsp70-z was expressed in *E. coli* JM109 cells transformed with pQE30/PfHsp70-z as previously described [11]. SDS-PAGE (12 %) and Western analyses (lower panel) of the expression and purification of recombinant PfHsp70-z, respectively. Lane M – Page ruler (Thermo Scientific) in kDa is shown on the left hand side; lane C - the total extract for cells transformed with a neat pQE30 plasmid; lane 0 – total cell extract transformed with pQE30/PfHsp70-1; pQE30/PfHsp70-z prior to IPTG induction; lanes 1 - 6, O/N – total cell lysate obtained 1 - 6 hours and overnight post induction. Lane P, S-pellet and soluble fractions obtained from the total lysate for cells transformed with pQE30/PfHsp70-1 or pQE30/PfHsp70-z, respectively; lane F; flow through; lane W —wash samples, and lane E—PfHsp70-z protein eluted using 500 mM imidazole. Lower panels: Western blot confirming expression, purification of PfHsp70-1 and PfHsp70-z based on using α-PfHsp70-1 and α-PfHsp70-z antibodies, respectively.
